# Long-term outcome of microwave ablation for benign thyroid nodules: Over 48-month follow-up study

**DOI:** 10.3389/fendo.2022.941137

**Published:** 2022-08-01

**Authors:** Jia-Rui Du, Wen-Hui Li, Cheng-Hai Quan, Hui Wang, Deng-Ke Teng

**Affiliations:** ^1^ Department of Ultrasound, China-Japan Union Hospital of Jilin University, Changchun, China; ^2^ Department Of Oncology, Hospital of Jilin Bureau of Geologic Exploration and Mineral Development, Changchun, China

**Keywords:** thyroid, benign nodules, thermal ablation, ultrasound, microwave ablation

## Abstract

**Objectives:**

The short-term effects of microwave ablation (MWA) for the treatment of benign thyroid nodules (BTNs) were satisfactory in previous studies. However, as a slowly progressing disease, the long-term efficacy of MWA for BTNs at present is not clear. Our study aim was to assess the long-term results of MWA for BTNs after a 48-month follow-up.

**Methods:**

From June 2015 to September 2017, 148 patients had 148 BTNs. All patients were from the China-Japan Union Hospital of Jilin University. Careful ultrasound examinations were performed 1 day, 1 month, 3 months, 6 months, 12 months, and every 6 months after MWA. The volume, volume reduction rate (VRR), recurrence rate of the ablated area and thyroid function were recorded.

**Results:**

The mean volumes of the 148 nodules were 15.6 ± 9.4 cm^3^ (range: 1.3-48.9 cm^3^) and 0.6 ± 0.6 cm^3^ (range: 0-3.5 cm^3^) before and 48 months after MWA, respectively, with a nodule VRR of 96.9 ± 2.5% (range: 90.4-100%). Two patients (1.35%) had recurrence after MWA. Compared with thyroid function before MWA, no significant variation was observed after MWA. Five patients experienced complications (3.38%): two patients (1.35%) had bleeding, two patients (1.35%) had ear pain and toothache during MWA, and one patient (0.68%) had hoarseness after MWA. No cases of oesophageal injury, tracheal injury, infection, skin burns, etc., were reported during or after MWA.

**Conclusions:**

Based on a long-term follow-up, MWA is an effective method for treating BTNs and is expected to be a potential first-line treatment.

## Introduction

In recent years, the detection rate of thyroid nodules has been as high as 65%, and more than 90% of nodules are benign (1, 2). Only a few of these benign nodules require treatment, due to nodules tend to grow and/or associated pressure and/or clinically symptomatic etc. Although surgery is a widely used and effective treatment regimen, it is traumatic, that results in a slow recovery and affects the appearance of the patient. Moreover, some patients must be placed on long-term thyroid hormone replacement therapy after surgery (3–5). Therefore, in recent years, several scholars have sought minimally invasive treatment (MIT) methods to treat benign thyroid nodules (BTNs).

One such method is ultrasound (US)-guided percutaneous microwave ablation (MWA) (6). MWA is a promising thermal ablation technique that has been used in tumours of the liver and kidney (7, 8) due to its continuously higher intratumor temperature and greater ablation range in a shorter ablation time (9, 10). In recent years, the short-term (less than 12 months) effects of MWA for the treatment of BTNs have been verified by some scholars with encouraging results, with a volume reduction rate (VRR) that ranges from 45.99% to 85.97% (6, 9, 11). However, as a slowly progressing disease (12), the real efficacy of MWA treatment for BTNs should be observed in a long-term follow-up of more than 12 months. Therefore, this study aimed to clarify the efficacy of MWA for the treatment of BTNs in a follow-up of at least 48 months.

## Materials and methods

### Study oversight

This retrospective study was approved by the Ethics Committee of the China-Japan Union Hospital of Jilin University, and all patients signed an informed consent form before undergoing MWA. All patients were confirmed to have BTNs by core-needle biopsy (CNB).

### Patients

The inclusion criteria were as follows (met all of the criteria) (1): benign nodules with once clear pathological result by CNB (Bethesda category: class II, benign), US imaging findings without malignant suspicious features (2); the longest diameter of the lesion is ≥2 cm (3); large nodules (the largest diameter > 4 cm) causing compressive or structural symptoms or psychological anxiety or that affected the appearance of the patient (4); only a single nodule of the thyroid gland (5); refusal to undergo surgical treatment or intolerance for surgery; and (6) normal thyroid functionality.

Patients must meet one of the following exclusion criteria (1): no clear pathological diagnosis or inaccurate CNB results (Nondiagnostic or Unsatisfactory; Atypia of Undetermined Significance or Follicular Lesion of Undetermined Significance; Follicular Neoplasm or Suspicious for a Follicular Neoplasm or Specify if Hurthle cell type) (2); pregnancy or lactation (3); severe abnormal blood coagulation (4); severe abnormal heart or lung function causing inability to tolerate MWA (5); nodules accompanied by macrocalcifications (>1 cm); and (6) infraclavicular goiters.

From June 2015 to September 2017, 153 patients with 153 lesions at our hospital underwent ultrasound-guided MWA for the treatment of BTN. Five patients with 5 lesions were lost to follow-up, and the remaining 148 patients with 148 lesions were ultimately included in this study. Among them, there were 33 males and 115 females with an average age of 41.2 ± 10.7 years (range: age 25-72 years).

### Equipment

A Siemens S2000 (Siemens Mountainview, USA) and MINDRAY^®^ DC-8Exp (MINDRAY, Shenzhen, China) colour Doppler ultrasonic diagnostic instrument was employed for ultrasonic image acquisition and MWA guidance. The probe was a 4-9 MHz linear array probe for detecting the superficial organs. An ECO-100A1 microwave treatment instrument (YIGAO Microwave System Engineering Co., Ltd., Nanjing, Jiangsu Province, China) and ECO-100AI3 superficial organ ablation needle (16 G, total length: 10 cm, microwave transmitter away from the shaft tip: 3 mm) were used for MWA.

### Pre-MWA procedures

Image collection included observing and recording the size, location, composition, echogenicity, margin, shape, echogenic foci, blood flow, etc., of the lesion. All images were collected by a senior doctor (with more than 10 years of experience in thyroid ultrasound examination). Contrast-enhanced ultrasonography (CEUS) was performed before MWA to obtain the scope and blood supply of the nodules and to design a corresponding MWA plan, which was designed by two experienced doctors (with more than 5 years of experience in MWA). The volume of the lesion was calculated using V =πabc∕6 (V: volume, a: maximum diameter, b and c: the other two perpendicular diameters). A thyroid function test was performed before MWA and at 1 month, 6 months, 12 months, and every 12 months thereafter.

Symptom and cosmetic scores and the Hospital Anxiety and Depression Scale (HADS) were evaluated before and after MWA. We used a visual analogue scale (with scores ranging from 0 to 10) to evaluate neck compression symptoms and dysphagia. The experienced physician evaluated the cosmetic appearance with a score from 1 to 4: 1: no palpable mass; 2: palpable but invisible mass; 3: cosmetic problems when swallowing; 4: cosmetic problems (6, 11). Patients with psychological anxiety about their disease were asked to complete the Anxiety Subscale (HADS-a) questionnaire of the Hospital Anxiety and Depression Scale (HADS), which was used to assess the degree of anxiety. The HADS-a consists of 7 items pertaining to anxiety (with scores ranging from 0 to 21). If the anxiety subscale values exceed seven, anxiety is indicated in the patients (13, 14).

### MWA procedure

The patient was laid supine with the neck fully exposed, and local infiltration anaesthesia with 1% lidocaine was administered. For lesions close to high-risk areas, e.g., near the trachea, oesophagus, recurrent laryngeal nerve and neck vasculature, normal saline was used as an isolation fluid to separate the high-risk area from the lesion to prevent damage to the surrounding important organs or tissues (15–17).

According to Teng’s puncture method, a 16 G sharp needle 4 cm in length was used to puncture the skin. When the needle was pulled out, a tunnel was created, through which the antenna was inserted and placed into the thyroid nodule by US guidance (18). On the MWA instrument, ablation mode was initiated with 25 W-30 W power. Using moving-shot techniques (19) during MWA, according to the volume, location and blood supply of the nodule, ablation was performed layer by layer from the upper pole to the lower pole or from the lower pole to the upper pole of the thyroid, following the “from far to near” or “from deep to shallow” principle (19). When the MWA process was performed near the recurrent laryngeal nerve, the operator verbally communicated intermittently with the patient to quickly detect any hoarseness. During the MWA process, the isolation fluid should be quickly replenished if it is absorbed.

A CEUS examination was performed immediately after MWA. If the ablation range was satisfactory, i.e., the “black hole” without contrast filling completely covered the tumour, then the ablation was considered complete; if the ablation range was not satisfactory, i.e., CEUS showed contrast filling the nodules, then supplemental ablation was performed.

### Follow-up

Careful ultrasound examinations were performed at 1 day, 1 month, 3 months, 6 months, 12 months, and then every 6 months after MWA. The volume and volume reduction rate (VRR) of the MWA area were observed and recorded using the following formula: VRR (%) = ([initial volume–final volume] × 100%)/initial volume. Recurrence was defined as an abnormal nodular echo found in the internal or marginal ablation area, a gradually growing nodular echo or abundant blood flow signals inside the nodule during follow-up; and then, a BTN was confirmed by CNB.

### Statistical analysis

SPSS 20.0 was used for statistical analysis. The size of the nodule and the age of the patient were described using the mean ± standard deviation (SD). The volume changes before and after MWA were measured by the paired samples *t*–test.

## Result

### Lesion characteristics

A total of 148 lesions were confirmed by CNB pathology to be BTNs. Among them, 81 were located in the right lobe, and 67 were located in the left lobe. The average maximum diameter of the nodules was 3.6 ± 0.8 cm (range: 2.0-5.8 cm), and the mean volume was 15.6 ± 9.4 cm^3^ (range: 1.3-48.9 cm^3^). Of the nodules, 46 (31.1%), 95 (64.2%), and 7 (4.7%) had volumes ≤10, 10–30, and ≥ 30 cm^3^, respectively. The mean ablation time was 456 ± 318 s. The baseline characteristics of the BTNs are presented in **Table 1**.

**Table 1:** The clinical features of patients and nodules.

Variable	Datum
No. of patients	148
Mean age (years)^*^	41.2 ±10.7 (25-72)
Gender	
Female	115 (77.7%)
Male	33 (22.3%)
No. of nodules	
≤ 10 cm^3^	46 (31.1%)
10–30 cm^3^	95 (64.2%)
≥ 30 cm^3^	7 (4.7%)
Mainly solid nodules	100 (67.6%)
Mainly cystic nodules	48 (32.4%)
Mean nodules diameters (cm)^*^	3.6±0.8 (2.0-5.8)
Mean nodules volumes (cm^3^)^*^	15.6±9.4 (1.3-48.9)
Growth locations of nodules	
Left lobe	67 (45.3%)
Right lobe	81 (54.7%)
symptom scores	4.1±1.3 (0-8)
cosmetic scores	2.1±0.8 (1-4)
Mean ablation time (s)	456±318

^*^Data are means ± standard deviation; data in parentheses are ranges.

### MWA procedure and complications

During MWA, all 148 patients tolerated the local anaesthesia well, and none required other analgesic drugs after MWA.

Five patients (3.38%) had complications in the peri-MWA period: major complications (0.68%) were observed in one patient (one with hoarseness), and minor complications (2.70%) were observed in four patients (two with bleeding and two with earache and toothache). No cases of oesophageal injury, tracheal injury, infection, skin burns, etc., were reported during or after MWA.

Of the two patients (1.35%) who had bleeding during MWA, 5 minutes of compression stopped the bleeding for one but not for the other, as confirmed by colour Doppler ultrasound at the bleeding site. For the other patient, the bleeding site was burned for 1 minute with an ablation power of 40 W, which successfully stopped the bleeding. None of the patients who had bleeding required surgical haemostasis.

Two patients (1.35%) experienced earache or toothache on the same side as the lesion during MWA, and the pain was relieved within two hours after MWA.

One patient (0.68%) had hoarseness after MWA and recovered 3 months after MWA. The nodule in this patient was larger than that in the other patients, measuring 5.8 cm×3.5 cm×4.6 cm, with a volume of 48.9 cm^3^, and was located adjacent to the dorsal thyroid gland.

Two patients (1.35%) had recurrence after MWA. Two recurrent nodules showed regrowth at the marginal region of the previous nodule at the 18-month and 24-month follow-ups. Both of them were diagnosed with a BTN by CNB and underwent a second MWA procedure. After the secondary procedure, two nodules were decreased in size and did not recur during the follow-up period, which continued for more than 24 months and 30 months.

### Follow-up

**Figure 1:**
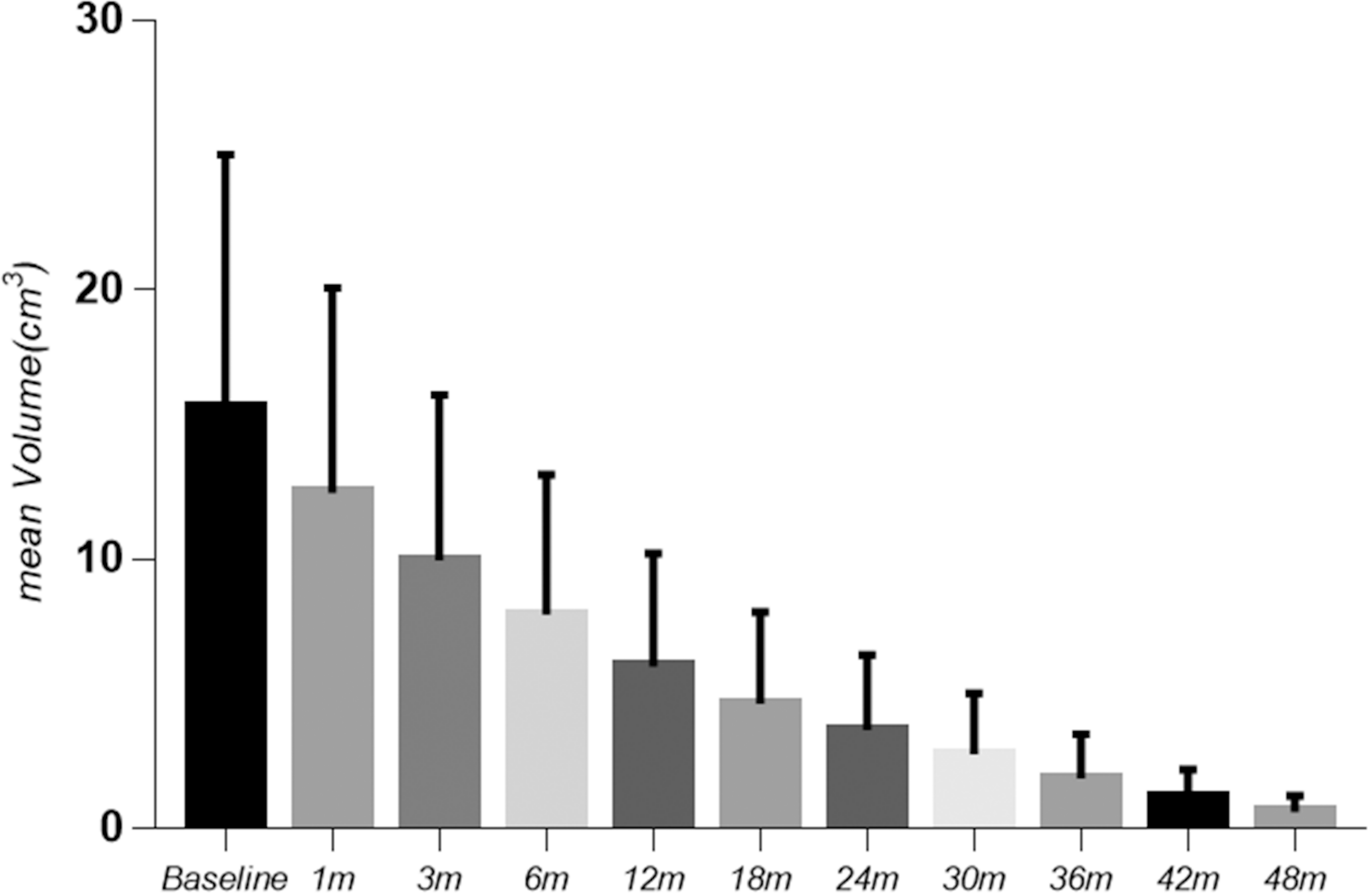
Variation in the mean volume before and after MWA at each follow-up point.

**Figure 2:**
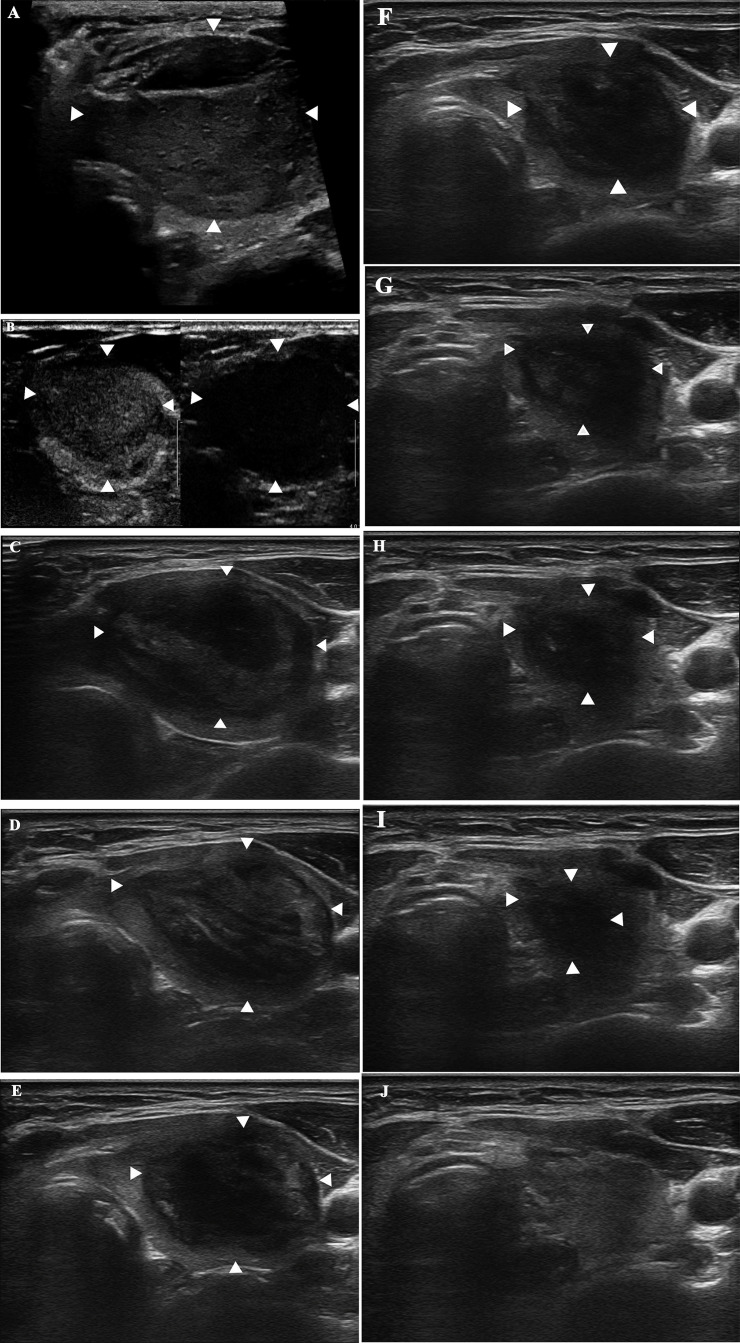
A 52-year-old woman with a diagnosis of a benign thyroid nodule in the left thyroid lobe. The images show the nodule before and during follow-up after MWA. **(A)** Before MWA, the nodule measures 4.6 cm × 3.1 cm × 3.5 cm in size and 26.1 cm^3^ in volume. **(B)** CEUS shows uniform low enhancement of the nodule before MWA. **(C)** 1 month after MWA, the ablation area measures 4.4 cm × 2.8 cm × 3.3 cm in size and 21.3 cm^3^ in volume. **(D)** 3 months after MWA, the ablation area measures 3.8 cm × 2.2 cm × 3.3 cm and 14.4 cm^3^ in volume. **(E)** 6 months after MWA, the ablation area measures 3.4 cm × 2.1 cm × 2.3 cm and 8.6 cm^3^ in volume. **(F)** 12 months after MWA, the ablation area measures 3.0 cm × 1.8 cm × 2.0 cm and 5.7 cm^3^ in volume. **(G)** 18 months after MWA, the ablation area measures 2.5 cm × 1.3 cm × 1.5 cm and 2.6 cm^3^ in volume. **(H)** 24 months after MWA, the ablation area measures 1.7 cm × 1.0 cm × 1.4 cm and 1.2 cm^3^ in volume. **(I)** 30 months after MWA, the ablation area measures 1.3 cm × 0.6 cm × 1.0 cm and 0.4 cm^3^ in volume. **(J)** 36 months after MWA, the ablation area has vanished.

The follow-up duration of the 148 patients was more than 48 months. 48 months after MWA, the mean volume of the 148 nodules significantly decreased from 15.6 ± 9.4 cm^3^ (range: 1.3-48.9 cm^3^) to 0.6 ± 0.6 cm^3^ (range: 0-3.5 cm^3^) (*P <*0.0001) (**Figure 1**), with a VRR of 96.9 ± 2.5% (range: 90.4-100%) (**Figure 2**). At 48 months, 44 nodules (29.7%) were completely absorbed. The changes in the volume of the nodules before MWA and at each follow-up time point are summarized in **Table 2**. Different VRRs were observed in different subgroups at the 48-month follow-up **(** **Table 3** **)**. For small nodules with a volume ≤ 10 cm^3^, the VRR at the 48-month follow-up was 99.1 ± 2.0%, and for medium nodules with a volume of 10–30 cm^3^, the VRR at the 48-month follow-up was 95.9 ± 1.9%. For large nodules with a volume of ≥30 cm^3^, the VRR at the 48-month follow-up was 94.2 ± 1.6%.

**Table 2:** Mean volume, volume reduction rate and absorption rate of the nodules after MWA.

Time	Mean volume of ablation area	Mean volume reduction rate (%)		Completely absorption rate of nodules (%)		*P* value(vs. volume before MWA)	
Baseline	15.6±9.4		–	–	–	
1 month	12.5±7.6		20.9±7.3	–	0.000	
3 months	9.6±6.1		38.1±10.4	–	0.000	
6 months	8.0±5.2		52.8±12.8	–	0.000	
12 months	6.0±4.2		66.3±12.9	2.0	0.000	
18 months	4.6±3.4		75.3±12.0	9.5	0.000	
24 months	3.3±2.8		81.5±10.9	14.2	0.000	
30 months	2.5±2.3		87.1±9.3	18.9	0.000	
36 months	1.8±1.6		91.0±7.0	23.0	0.000	
42 months	1.2±1.1		94.4±4.4	27.0	0.000	
48 months	0.6±0.6		96.9±2.5	29.7	0.000	

Data are means ± standard deviation.

**Table 3:** Nodule characteristics and the effects of MWA at different sizes.

	Nodule Volume		
	≤ 10 cm^3^	10–30 cm^3^	≥ 30 cm^3^
No. of nodules	46	95	7
Nodule volume (cm^3^)			
Baseline	3.5±2.5	19.2±5.3	37.3±5.6
1 month	3.9±2.2^*^	15.4±4.5^*^	29.9±2.8^*^
12 months	1.4±1.4^*^	7.5±2.6^*^	15.8±2.1^*^
24 months	0.6±0.8^*^	4.7±1.8^*^	9.5±1.9^*^
36 months	0.2±0.5^*^	2.4±1.1^*^	5.7±1.6^*^
48 months	0.1±0.1^*^	0.8±0.4^*^	2.2±0.7^*^
VRR (%)			
1 month	21.7±9.1	20.2±6.2	19.1±6.6
12 months	75.4±16.9^#^	61.0±6.9^#♦^	57.4±4.6^#♦^
24 months	91.2±10.2^#^	75.4±7.1^#♦^	74.6±3.6^#♦^
36 months	97.5±5.4^#^	87.6±4.8^#♦^	84.8±2.7^#♦^
48 months	99.1±2.0^#^	95.9±1.9^#♦^	94.2±1.6^#♦^

Data are means ± standard deviation.

Abbreviation: VRR, volume reduction rate.

^*^P < 0.05 versus baseline values respectively, ^#^P < 0.05 versus VRR (%) at 1-month follow-up after ablation, respectively, ^♦^P < 0.05 versus VRR (%) at ≤ 10 cm^3^ group respectively.

All 148 patients underwent thyroid function examination before MWA and at 1 month, 6 months, 12 months, and then every 12 months after MWA, and there was no significant variation in thyroid function in any of the patients after MWA compared with thyroid function before MWA.

### Symptomatic, cosmetic and anxiety problems

**Figure 3:**
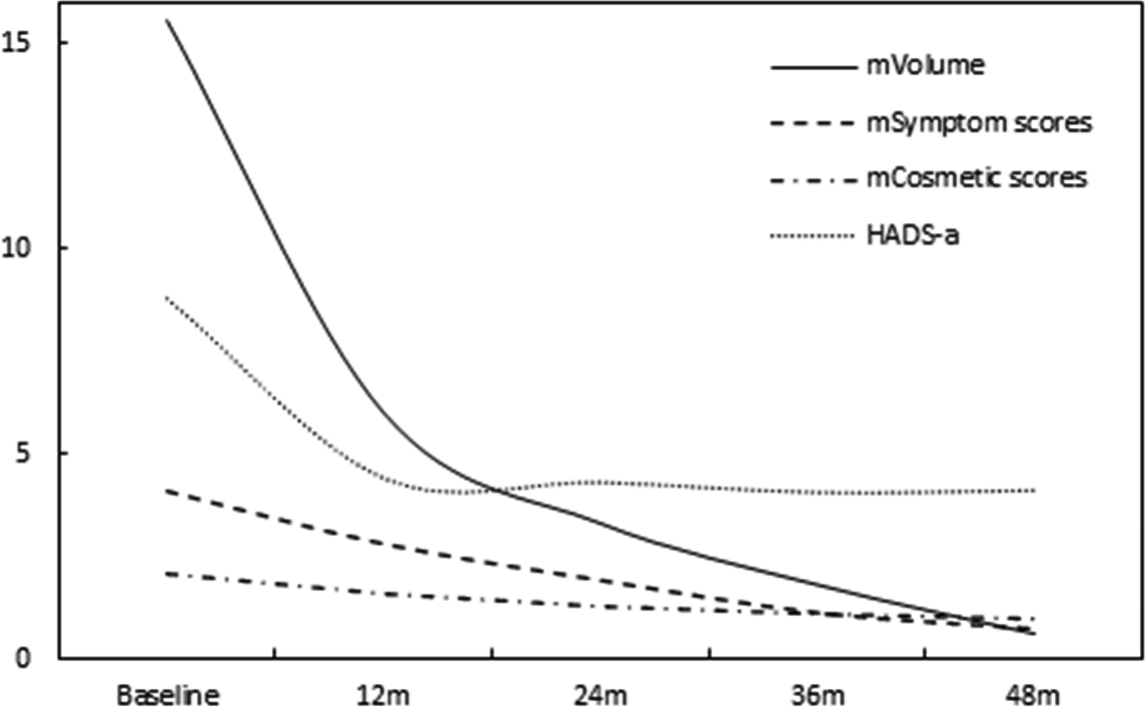
At each follow-up time point, the symptoms and cosmetic scores and HADS-a varied with thyroid nodule shrinkage.

The symptom and cosmetic scores were 4.1 ± 1.3 (range: 0-8) and 2.1 ± 0.8 (range: 1-4) before MWA, respectively. 48 months later, it was significantly decreased to 0.7 ± 0.5 (range: 0-2) (*P*<0.0001) and 1.0 ± 0.2 (range: 1-2) (*P*=0.0002), respectively. Prior to MWA, 27.0% (40/148) of the patients complained of psychological anxiety. At the 48-month follow-up, the HADS-a scores of those patients significantly decreased from 8.8 ± 1.3 (range: 8-13) to 4.1 ± 1.8 (range: 1-7) (*P*<0.0001). All of the discomfort and anxiety problems mentioned above were completely resolved at the 48-month follow-up. (**Figure 3**)

## Discussion

In this study, 148 nodules in 148 patients were treated with MWA, and the patients were followed-up for 48 months. The long-term follow-up results of the study showed that MWA was effective and safe for the treatment of BTNs. In previous research (6, 9, 11, 20, 21), the follow-up duration after MWA was 6-12 months, and the mean VRR of the nodules was 45.99%-85.97%. However, BTNs are slow progressing, and a short-term follow-up may not be enough to accurately determine recurrence after MWA (12).

In this study, MWA was used to treat BTNs and achieved good therapeutic effects according to the long-term follow-up. After the 48 months of follow-up after MWA, the nodule volume was significantly reduced, with a VRR of 96.9 ± 2.5% (range: 90.4-100%). Similar to Luo et al. (22) and Liu et al. (23), the mean VRR of the nodules was 93.2% and 97.1%, respectively, in two 3-year follow-ups. Meanwhile, nodule shrinkage was also accompanied by improvements in symptoms and cosmesis, and MWA for the treatment of BTNs in this study significantly alleviated the patients’ symptoms and cosmetic problems at the time of the last follow-up. Interestingly, although the previous literature (24–28) had a shorter follow-up period than our study, we found similar results: the volume reduction rapidly increased in the first 6-12 months, and the symptoms and cosmetic problems improved as the nodule volume decreased.

After 48 months of follow-up after MWA, among all 148 nodules, 44 nodules were completely absorbed (13 were mainly solid nodules and 31 were mainly cystic nodules), and the complete disappearance rate was 29.7%. Similar to Yue’s study (27), the complete disappearance rate was 30.7%. However, 104 nodules were not completely absorbed among the cohort in our study. The mean VRR of these nodules was 95.3 ± 1.7% (range 90.4-98.7%) after more than 48 months of follow-up. The nodules (mean volume 19.69 ± 7.77 cm3) that were not completely absorbed in our study were larger (*P*<0.000), more solid and less cystic (*P*=0.004) than the nodules (mean volume 6.04 ± 4.63 cm3) that were completely absorbed. At the same time, Yue’s study (23, 27) also confirmed that mixed and cystic nodules were better absorbed after ablation. Therefore, we speculate that the volume and components of the nodules may be the main factors affecting their absorption after MWA.

Our study revealed a recurrence rate of 1.35% (2/148), which was lower than that in Cheng’s (11), Lim’s (29) and Deandrea’s (30) studies, in which the recurrence rates were 7.7% (51/664), 5.6% (7/126) and 4.1% (9/215), respectively. We hypothesize three reasons for the low recurrence rate after MWA in this study. First, the application of CEUS helped to quickly detect the presence of residual nodules after MWA and to minimize or avoid recurrence through timely supplemental ablation. Second, during MWA, the supplying blood vessel was usually located at the edge of the nodule; thus, the marginal area of the nodule should be fully ablated. Third, careful planning before MWA and strict implementation of the plan during MWA were crucial for preventing recurrence after MWA.

This study showed that MWA was safe for the treatment of BTNs. Five patients (3.38%) in this study had complications in the peri-MWA period, which was similar to Lim’s (29) study, and the overall complication rate in Lim’s study was 3.6% (4/111).

In our study, two patients (1.35%) experienced bleeding in the anterior neck space of the thyroid capsular, which may be caused by damage to the thyroid capsule and its surrounding blood vessels by the puncture path. During the MWA procedure in our study, the bleeding risk rate was lower than that in previous studies on MWA and RFA for the treatment of BTNs, such as studies by Liu et al. (10), Korkusuz et al. (31) and Deandrea et al. (32), who found bleeding risk rates of 3.4%, 7.1% and 15%, respectively. The lower bleeding risk rate in this study was attributed to the following reasons. First, careful observation with colour Doppler prior to puncture could avoid some bleeding, which was caused by damage to the small blood vessels during the puncture process. Second, colour Doppler ultrasound was used to locate the bleeding site quickly and accurately, and MWA could be used to perform haemostasis with 1 minute of ablation at a power of 40 W.

One patient (0.68%) experienced hoarseness that recovered after 3 months, which was lower than Zhi Xi’s (3) study (3.6%) and Cheng’s (11) report (5.8%), but is similar to that in Deandrea’s (32) study (0.4%). Our experience shows that the treatment of BTNs should comprise full ablation under the premise of safety. Therefore, when ablating BTNs, heat injury of the peripheral nerves should be avoided as much as possible. When the duration of ablation is too long, it is important to quickly replenish the isolation liquid to prevent a nerve injury (33–35).

Our study also demonstrated other advantages. The thyroid function of all 148 patients was reviewed 48 months after MWA, showing no significant variation compared with that before MWA. This shows that MWA does not cause hypothyroidism in patients, which is a very important advantage of MWA for BTNs.

This research also has some limitations. First, this research had a small sample size. Second, this study was conducted at a single centre; a multicentre study with a larger sample should be conducted in the future to further verify the results. Finally, although CNB was performed in all 148 nodules in this study to reduce the occurrence of false negatives, this cannot be completely avoided.

In conclusion, over 48-month follow-up, MWA for the treatment of BTNs is effective and safe and is expected to be a potential first-line treatment.

## Data availability statement

The raw data supporting the conclusions of this article will be made available by the authors, without undue reservation.

## Ethics statement

The studies involving human participants were reviewed and approved by Ethics Committee of the China-Japan Union Hospital of Jilin University. The patients/participants provided their written informed consent to participate in this study. Written informed consent was obtained from the individual(s) for the publication of any potentially identifiable images or data included in this article.

## Author contributions

HW and D-KT contributed to the study conception and design. The first draft of the manuscript was written by J-RD. Material preparation, data collection and analysis were performed by W-HL and C-HQ, and all authors commented on previous versions of the manuscript. All authors read and approved the final manuscript.

## Funding

This work was supported by the Department of Science and Technology of Jilin Province (Grant No. 20190303140SF), the Jilin Provincial Health and Family Planning Commission (Grant No. 2019SCZ027) and the China Postdoctoral Science Foundation (Grant No. 2019M661219).

## Acknowledgments

The authors would like to thank Xue-Qing Feng for her encouragement and support of this study.

## Conflict of interest

The authors declare that the research was conducted in the absence of any commercial or financial relationships that could be construed as a potential conflict of interest.

## Publisher’s note

All claims expressed in this article are solely those of the authors and do not necessarily represent those of their affiliated organizations, or those of the publisher, the editors and the reviewers. Any product that may be evaluated in this article, or claim that may be made by its manufacturer, is not guaranteed or endorsed by the publisher.

## Abbreviations

MWA, microwave ablation; BTNs, benign thyroid nodules; RFA, radiofrequency ablation; VRR, volume reduction rate; CNB, core-needle biopsy; CEUS, contrast-enhanced ultrasonography.
